# A novel approach to label bone marrow-derived mesenchymal stem cells with mixed-surface PAMAM dendrimers

**DOI:** 10.1186/s13287-019-1171-7

**Published:** 2019-02-28

**Authors:** Nikolas Munro, Bhairavi Srinageshwar, Firas Shalabi, Maria Florendo, Paulina Otero, Cassandra Thompson, Jordyn Kippe, Clayton Malkowski, Sydney Climie, Andrew N. Stewart, Rachel Kim, Joseph Zhou, Douglas Swanson, Gary L. Dunbar, Ajit Sharma, Julien Rossignol

**Affiliations:** 1 College of Medicine, Central Michigan University, Mount Pleasant, MI USA; 2 Field Neurosciences Institute Laboratory for Restorative Neurology, Mount Pleasant, MI USA; 3 Program in Neuroscience, Central Michigan University, Mount Pleasant, MI USA; 4 Department of Psychology, Central Michigan University, Mount Pleasant, MI USA; 5 Department of Chemistry & Biochemistry, Central Michigan University, Mount Pleasant, MI USA; 6 Field Neurosciences Institute, Saginaw, MI USA

**Keywords:** PAMAM dendrimer nanoparticles, Mesenchymal stem cells, Cell labeling technique, Cell transplantation, Hoechst, Imaging

## Abstract

**Background:**

Transplantation of mesenchymal stem cells has created enormous opportunities as a potential treatment for various diseases including neurodegenerative diseases. Given current techniques, such as Hoechst labeling, have safety and leakage issues, our study focused, as a proof-of-concept, on a new dendrimer-based technique for labeling these stem cells to ensure their efficacy and safety following transplantation into the brain of a healthy mice.

**Methods and results:**

The bone marrow-derived mesenchymal stem cells (BM-MSCs) were labeled using polyaminoamine (PAMAM) dendrimers following which their stemness based on their proliferation and differentiation ability were analyzed by gold standard methods. These labeled BM-MSCs were transplanted into the striatum of C57BL/6J mice and were tracked using in vivo imaging system (IVIS) and analyzed using tissue imaging, 2 weeks after transplantation. Our results showed that the dendrimer-labeled BM-MSCs were able to successfully maintain their stemness and were tracked in vivo following transplantation. Unlike Hoechst, we did not find the dendrimers to be leaking out of the cells and were very specific to the cells that up took the dendrimers. Moreover, no adverse events were found in the transplanted animals proving that this is a safer method.

**Conclusions:**

Labeling BM-MSCs using fluorescently tagged PAMAM dendrimers can be used as a potentially safe and efficient method for labeling cells, particularly stem cells, in vitro and in vivo following transplantation in rodents.

## Background

Mesenchymal stromal cells consist of heterogeneous population of adult cells that contain stem cells, known as mesenchymal stem cells (MSCs) having useful applications in regenerative medicine. They are multipotent stem cells that are defined by three main characteristics: plastic adherence, ability to naturally differentiate into a diverse set of tissues within the mesoderm lineage, and of self-renewal [1, 2]. Although these cells can be derived from umbilical cord, adipose tissue, and many other tissues [3], this study focuses only on bone marrow-derived MSCs (BM-MSCs), since they are the most predominant in the body. We have frequently used them in our laboratory for studying potential treatments of various neurodegenerative diseases and spinal cord injuries. We have previously shown that intrastriatal transplantation of BM-MSCs ameliorates the motor and cognitive deficits in different HD rodent models. Analysis of brain tissue showed an increase in brain-derived neurotrophic factor (BDNF), indicating that BDNF secretion by the transplanted MSCs helped the neurons to survive [4, 5]. Our lab also explored the use of genetically modified MSCs as a potential treatment for spinal cord injuries [6].

MSCs have the ability to differentiate into different lineages. From the mesoderm lineage, MSCs can be differentiated into osteoblasts (cells that provide the matrix for bones), adipocytes (or fat cells), and into chondrocytes (cells that secrete cartilage; [7]). Each of these lineages has potential applications in regenerative medicine. Adipocytes can be used for the integration of several tissues, tissue homeostasis, tissue regeneration, and the regulation of pathology of several diseases. Osteogenic differentiation of MSCs has also been shown to help in bone tissue engineering and cartilage repair for osteoarthritis [8].

Given the importance of MSCs in various neurological disorders and their therapeutic values of their differentiated forms, it is important to be able to track these cells following transplantations into the brain and other organs. The conventional method of labeling MSCs prior to transplantation is using bisbenzimide (Hoechst). However, there are major drawbacks of using bisbenzimide as a tool for labeling cells. One of its disadvantages is its preferential binding to DNA, particularly in apoptotic cells [9], potentially interfering with DNA replication during cell division [10–12] and inducing apoptosis [13]. Conover and Gwatkin observed that higher concentrations of bisbenzimide (greater than or equal to 10 μg H-33342/mL) were detrimental to mature mouse oocytes. Similarly, Adamski and colleagues (2007) demonstrated that bisbenzimide can affect cell differentiation by altering the complex between DNA and TATA box-binding protein and can function as a specific topoisomerase-I poison. Hoechst has also been demonstrated to be susceptible to photobleaching after radiation, limiting its use in certain experimental conditions [14]. Considering the potential problems with this organic compound, we were interested in finding alternative labeling methods for MSCs, including the use of polyaminoamine (PAMAM) dendrimers.

PAMAM dendrimers are highly branched nanomolecules widely researched for various applications. Dendrimers are mainly composed of a core, branches, and surface groups. Dendrimers are one of the smallest nanomolecules known, with its size determined by its generation. Each generation refers to how highly branched the dendrimer is, with G1 being the least branched having a diameter of ~ 1 nm while a G4 is more branched with a diameter of ~ 4 nm [15]. We have modified the conventional G4 100% amine (-NH_2_) surface dendrimers (composed of 64 surface amines per dendrimer) into novel G4 mixed-surface dendrimers (G4 90/10), which have 90% of their surface covered with hydroxyl groups (-OH) and only 10% of the surface composed of amine groups (6 surface amines per dendrimer). Reducing the amines on G4 100% NH_2_ surface dendrimers was necessary as previous studies have shown that cell toxicity is associated with surface amine charge density. It has been shown that dendrimers having cationic charge are more cytotoxic than dendrimers composed entirely of –OH [16–18]. We have shown that these dendrimers can be readily fluorescently tagged (using their surface amines) and were taken up by the neurons and glial cells in vitro. They were also taken up by neurons and glial cells in vivo, when injected intracranially into the striatum or systemically via the carotid artery [19].

We show in this report that MSCs labeled with dendrimers maintain their properties of proliferation and differentiation, establishing their potential use for regenerative medicine. Moreover, the dendrimer-labeled MSCs could be successfully tracked by in vivo imaging following transplantation into the striatum of C57BL/6J mice.

## Methods

### Dendrimer synthesis and labeling with FITC or Cy5.5

G4-90/10 dendrimers and their conjugates with fluorescein isothiocyanate (FITC) or cyanine 5.5 (Cy5.5) were synthesized as previously described [19]. We shall refer to these tagged dendrimers as D-FITC and D-Cy5.5.

### Animals

A total of 34 male and female C57BL/6J mice were used in this study (Jackson Laboratory, Bar Harbor, ME, USA). Thirty-one animals (12–15 weeks old) were used for transplantation and in vivo imaging studies while three animals (5 weeks old) were used for MSC extraction from the bone marrow (BM). The mice were split into four groups: mice transplanted with 800k Luc2-dTom MSCs labeled with D-Cy5.5 (*n* = 8), mice transplanted with vehicle control (HBSS; *n* = 7), mice transplanted with 800k Luc2-dTom MSCs alone (*n* = 8), and mice transplanted with 800k D-Cy5.5-labeled MSCs (*n* = 8).

All the procedures followed the guidelines of the Institutional Animal Care and Use Committee (IACUC) of Central Michigan University (16 August 2018, and was registered under the CMU IACUC protocol #18-23). The animals were housed in polycarbonate cages at 22 °C under 12-h light/12-h dark cycle. They were given access to food and water *ad libitum*.

### Bone marrow extraction from C57BL/6J animals

The BM was extracted from three 5-week-old C57BL/6J animals as previously described [20]. Briefly, the animals were euthanized and the BM was extracted from their fibula. The freshly extracted BM cells (P0) were plated in alpha-MEM media containing 10% fetal bovine serum (FBS), 10% horse serum (HS), and 1% penicillin and streptomycin (Gibco Waltham, MA, USA). The cells were incubated at 37 °C for 48 h to remove non-adherent cells, following which the cell culture media was aspirated and fresh media was added.

### Cell passage

Once the P0 cells attained 85% confluency, they were passaged seven times (P1–P7). The cell passaging protocol was adapted from our previous study [5].

### In vitro

#### Uptake of D-FITC by bone marrow-derived MSCs

Various concentrations of D-FITC were added to the P7 BM-MSCs, and the cells were incubated at 37 °C. The cells were subsequently passaged a few times until no fluorescence from the labeled cells was observed under the microscope. Following dendrimer uptake by P7 cells, the existing media was aspirated and fresh media was added. Prior to dendrimer uptake, the MSCs were labeled with PKH26 according to the manufacturer’s protocol (Sigma-Aldrich, St. Louis, MO, USA). The labeled cells were fixed with 4% paraformaldehyde (PFA; Thermo Fisher Scientific, Waltham, MA, USA), mounted, and imaged using an Olympus BX50 upright confocal microscope. As controls, unlabeled MSCs and MSCs with FITC alone were used.

#### Live imaging of cellular uptake of D-Cy5.5 by BM-MSCs

Time-lapse images were acquired using a Leica DMI6000B inverted microscope with a × 40 dry objective lens. MSCs were plated on a 35-mm glass bottom petri dish (In Vitro Scientific, CA, USA) and incubated during imaging in a PECON incubator S incubation system and PECON heating insert P (PeCon GmbH, Erbach, Germany). MSCs were maintained at 5% CO_2_ and 20% O_2_ at 37 °C in an incubator set by a controller. The D-Cy5.5 at 4 mg/mL was added to different chambers containing MSCs. The bright-field and fluorescent images were captured with an ORCA-R2 camera (Hamamatsu, Japan) over 24 h. Captured images were processed and analyzed using LAS AF software (Leica, Germany).

#### Passaging D-FITC-labeled MSCs

Once cellular uptake was confirmed, the unlabeled P6 MSCs were passaged to obtain P7 MSCs and D-FITC were added to them at 4 mg/mL and incubated at 37 °C for 30 min. No other cellular stain was used to label the MSCs. Following 30 min incubation, fresh media was added and the cells were allowed to grow. Once the cells reached 85% confluency, they were passaged similar to the passaging of unlabeled cells as described previously [5]. In between every passage, the cell culture media was collected and used for further experiments, as described in the later sections. Before and after every passage, the MSCs were imaged using the Zeiss Observer inverted microscope to analyze the presence and biodistribution of dendrimers in MSCs. The cells were passaged until no D-FITC was observed in the cells. Appropriate control cells were used to which Hank’s balanced salt solution (HBSS; Gibco) was added.

#### Characterization of D-FITC-labeled MSCs

In between every passage, a population of cells was plated on 25-mm glass coverslips coated with 0.2 mg/mL of poly-l-lysine (PLL; Sigma-Aldrich). Once the cells reached 85% confluency, the cells were characterized for stem cell antigen-1 (Sca-1) marker as mouse MSCs predominantly express this marker [21]. The cell culture media were aspirated, and the cells were fixed on a coverslip using 4% PFA for 5 min. The coverslips were rinsed with 0.01 M phosphate-buffered saline (PBS), Rat anti-Sca-1 antibody (1/500; Sca-1; ab5131, Abcam, Cambridge, UK) was added, and the coverslips were incubated for 2 h on ice. The coverslips were rinsed again, and appropriate secondary antibody (AlexaFluor 594; Thermo Fisher Scientific) was added and incubated for 2 h on ice. The nucleus was stained with Hoechst 33342 (Thermo Fisher Scientific; 1/500) for 5 min on ice and rinsed twice with 1× PBS and once with phosphate buffer (PB). The coverslips were then mounted and imaged using the Zeiss Observer inverted microscope to analyze the presence of Sca-1 on D-FITC-tagged MSCs.

#### Analysis of cell culture media between every passage to assess leakiness of dendrimer following cell proliferation

UV-visible spectra of FITC and D-FITC in various solvents (media and PBS) were obtained on a Cary 1 UV-visible spectrophotometer (Varian, Palo Alto, CA) at wavelengths between 200 and 800 nm.

#### Characterization of D-Cy5.5-labeled MSCs

Coverslips, pre-coated with 0.2 mg/mL of poly-l-lysine, were placed in three 6-well plates and incubated overnight. Forty thousand MSCs were plated into each well with the pre-coated coverslips. The cells were incubated with D-Cy5.5 at 4 mg/mL concentration for 30 min, and HBSS was added as a control. Following incubation, the cells were washed twice with 0.01 M PBS. The experiments were done in duplicates.

#### Differentiation of the D-Cy5.5-labeled MSCs into adipocytes, osteoblasts, and chondrocytes

Two milliliters of specific differentiation media was added to each of the three plates having D-Cy5.5-labeled MSCs and controls. The adipocyte differentiation media consisted of DMEM, 10% FBS 1% penicillin–streptomycin (Gibco), 250 nM dexamethasone (Sigma-Aldrich), and 0.5 mM 30-isobutyl-1-methylxanthine (Sigma-Aldrich). The osteoblast differentiation media contained DMEM, 10% FBS, 1% penicillin–streptomycin, 50 mg/mL l-ascorbic acid (Sigma-Aldrich), 10 mM beta-glycerophosphate (Sigma-Aldrich), and 100 nM dexamethasone (Sigma-Aldrich). The chondrocyte differentiation media included 10 ng/mL of transforming growth factor (Sigma-Aldrich), 10 ng/mL basic fibroblast growth factor (bFGF; PrepoTech, Rocky Hill, NJ), 50 mg/mL ascorbic acid bisphosphate (Sigma-Aldrich), and 100 nM dexamethasone. Every day for 3 weeks, half of the media was changed with fresh media. At the end of the 3-week period, the D-Cy5.5 differentiated cells were washed twice with 0.01 M PBS and then fixed with 4% PFA (Thermo Fisher Scientific, Waltham, MA, USA).

#### Staining for D-Cy5.5-labeled MSCs and control MSCs for osteoblasts

Osteoblasts were stained with Alizarin red solution which consisted of 2 g Alizarin Red S (Sigma-Aldrich) diluted in 100 mL distilled water, adjusted to a pH of ~ 4.2 with HCl, and filtered. Enough solution was added to cover the cells attached to the coverslips. The cells were incubated at room temperature for 45 min and washed four times with distilled water. The cells remained in a 0.01 M PBS solution until the coverslips were mounted.

#### Staining for D-Cy5.5-labeled MSCs and control MSCs for adipocytes

To stain the adipocyte cells, Oil Red O solution was used. The solution was prepared by adding 0.5 g of Oil Red O (Sigma-Aldrich) dissolved in 100 mL of isopropanol in a water bath that has been heated to 37 °C. Thirty milliliters of this solution was added in 20 mL of distilled water. It was allowed to stand for 10 min and subsequently filtered. The adipocyte cells were first washed twice with distilled water followed by 60% isopropanol. After incubating for 5 min, the isopropanol was removed. Oil Red O Working Solution was then added to cover the cells attached to the coverslip and incubated for 10 min. The cells were washed with tap water followed by incubation with hematoxylin (Sigma-Aldrich) for 1 min. The cells were again washed five times with tap water and were mounted.

#### Staining for D-Cy5.5-labeled MSCs and control MSCs for chondrocytes

The chondrocytes were stained with Alcian Blue. The stain was prepared with adding 1 g Alcian Blue (Sigma-Aldrich) to 100 mL of 3% acetic acid (Sigma-Aldrich). The pH of the solution was adjusted to 2.5 with acetic acid. After the cells were fixed, they were washed thrice with 0.01 M PBS, then stained and incubated for 2 h. The cells were then rinsed with 0.1 M HCl once. The cells were then washed twice with PBS after which they were mounted. The undifferentiated control cells were also stained by the same procedure as mentioned above. The stained differentiated and undifferentiated MSCs were imaged using the Zeiss Observer inverted microscope and Zeiss Axio Imager M1 microscope (Carl Zeiss AG).

### In vivo

#### MSCs engineered with Luc2-dTom

Although all in vitro experiments were done with genetically unmodified MSCs, the in vivo experiments were carried out with genetically modified BM-MSCs so that the cells could be tracked using an in vivo imaging system (IVIS). The MSCs were modified to express a fusion reporter gene that codes for the firefly luciferase protein through a P2A sequence to tandem-dimer tomato (tdTomato; tdT). This was virally transduced into a population of MSCs [pCDH-EF1-Luc2-P2A-tdTomato was a gift from Kazuhiro Oka (Addgene plasmid # 72486), known as Luc2-dTom]. These tdT-expressing MSCs were transplanted bilaterally into the striatum of mice and tracked using an IVIS (Perkin Elmer, Waltham, MA) and observed via the Zeiss Axio Imager M1 microscope (Carl Zeiss AG). To ensure that the genetically modified BM-MSCs did not possess different properties than the unmodified BM-MSCs, an in vitro comparison tri-lineage differentiation (gold standard test for analyzing the stemness of MSCs) was performed between both cell lines.

#### Groups

The animals were randomly divided into four groups (1) animals transplanted with 800k Luc2-dTom MSCs labeled with D-Cy5.5, (2) animals that received the vehicle control (HBSS), (3) animals transplanted with 800k Luc2-dTom MSCs alone, and (4) animals transplanted with 800k D-Cy5.5-labeled MSCs.

#### Transplantation of Cy5.5-labeled MSCs

The Luc2-dTom-engineered MSCs were labeled with D-Cy5.5 as described above. The MSCs were prepared for transplantation into C57BL/6J animals, and the transplantation procedure was followed as previously described [5]. The animals were anesthetized with 2% isoflurane gas and 0.8% oxygen. The head of each mouse was shaved from the line between ears to just behind the eyes. Then, each anesthetized mouse was placed into the stereotaxic device (Kopf Instruments, Tujunga, CA). The surgical site was then cleaned with chlorhexidine (Molnycke Healthcare, Norcross, GA). A midline incision was then made on the scalp, and the skin was retracted to either side. Burr holes were drilled directly over the striatum. The coordinates of the bregma were documented, and the micro-syringe was moved accordingly (anterior + 0.5 mm, lateral + 1.75 mm, with the tooth bar set at − 3.3 mm). The MSCs or HBSS were loaded into a 10-μL Hamilton micro-syringe. Every mouse received bilateral injections of MSCs or HBSS at a consistent rate of 0.33 μL/min. The left hemisphere was then injected with 200,000 cells, 2.5 mm ventral to the dura. This was followed by a 3-min rest period. The micro-syringe was then moved 0.1 mm dorsally, and 200,000 more cells were injected into the striatum. This was again followed by a 3-min rest period. The syringe was then slowly withdrawn and re-positioned over the bregma where the coordinates for the striatum of the contralateral hemisphere were determined (anterior + 0.5 mm, lateral − 1.75 mm) and the procedure was repeated. Each hemisphere received a total of 400,000 cells (total of 800,000 cells/animal), while the vehicle control group received 2 μL HBSS. Incisions were closed by silk, non-absorbable sutures. A lidocaine ointment was liberally applied to the incision site. Following surgeries, the mice were monitored in recovery cages until they were fully alert and recovered. They were then placed back into their home cage and kept in an environment that was heated to body temperature and monitored until they were awake and alert. Once alert, the animals were placed back into their home environment where they were monitored and weighed for the next 5 days post-transplantation.

#### Tracking D-Cy5.5-labeled MSCs using in vivo imaging system

In vivo Imaging was performed at week 1 and week 2 for the following transplantation groups: (1) D-Cy5.5-labeled Luc2-dTom MSCs; (2) D-Cy5.5-labeled MSCs; (3) Luc2-dTom MSCs and (4) mice given HBSS. At 1 and 2 weeks post-transplantation, bioluminescent images and fluorescence brain images of animals were acquired with the IVIS Lumina LT In vivo Imaging System using Living Image 4.5.2 software (RRID:SCR_014247). This device is an optical imaging technology to facilitate non-invasive longitudinal monitoring of disease progression, cell trafficking, and gene expression patterns in living animals. Prior to placing the animals in the machine, the animals were anesthetized with 2% isoflurane gas and 0.8% oxygen. d-Luciferin sodium salt (Goldbio, St. Louis, MO, USA) was administered via intraperitoneal injections at 75 mg/kg body weight to detect the bioluminescence signal from the Luc2-dTom MSCs and fluorescent signal from D-Cy5.5 from MSCs (at 675 nm wavelength).

#### Slicing and histology

The mice were sacrificed 1 and 2 weeks post-transplantation. Following rapid extraction, the brains were suspended in 4% PFA for 24 h at 4 °C, after which they were transferred to 30% sucrose for 48 h at 4 °C. The brains were then frozen using methyl butane (Sigma-Aldrich) and stored at − 80 °C until needed for further evaluation. The tissue was then sectioned at 30 μm on a cryostat (Vibrotome UltraPro 5000). The tissue collected spanned the entire striatum. The slices were then mounted on positively charged microscope slides (Globe scientific Inc., Paramus, USA). Images were collected with a Zeiss Axio Imager M1 microscope (Carl Zeiss AG).

#### Ex vivo imaging

The slices of the brains were also imaged (ex vivo) in the IVIS Lumina LT In vivo Imaging System, using the Living Image 4.5.2 software (RRID:SCR_014247).

## Results

### Dendrimer synthesis and labeling

**Fig. 1:**
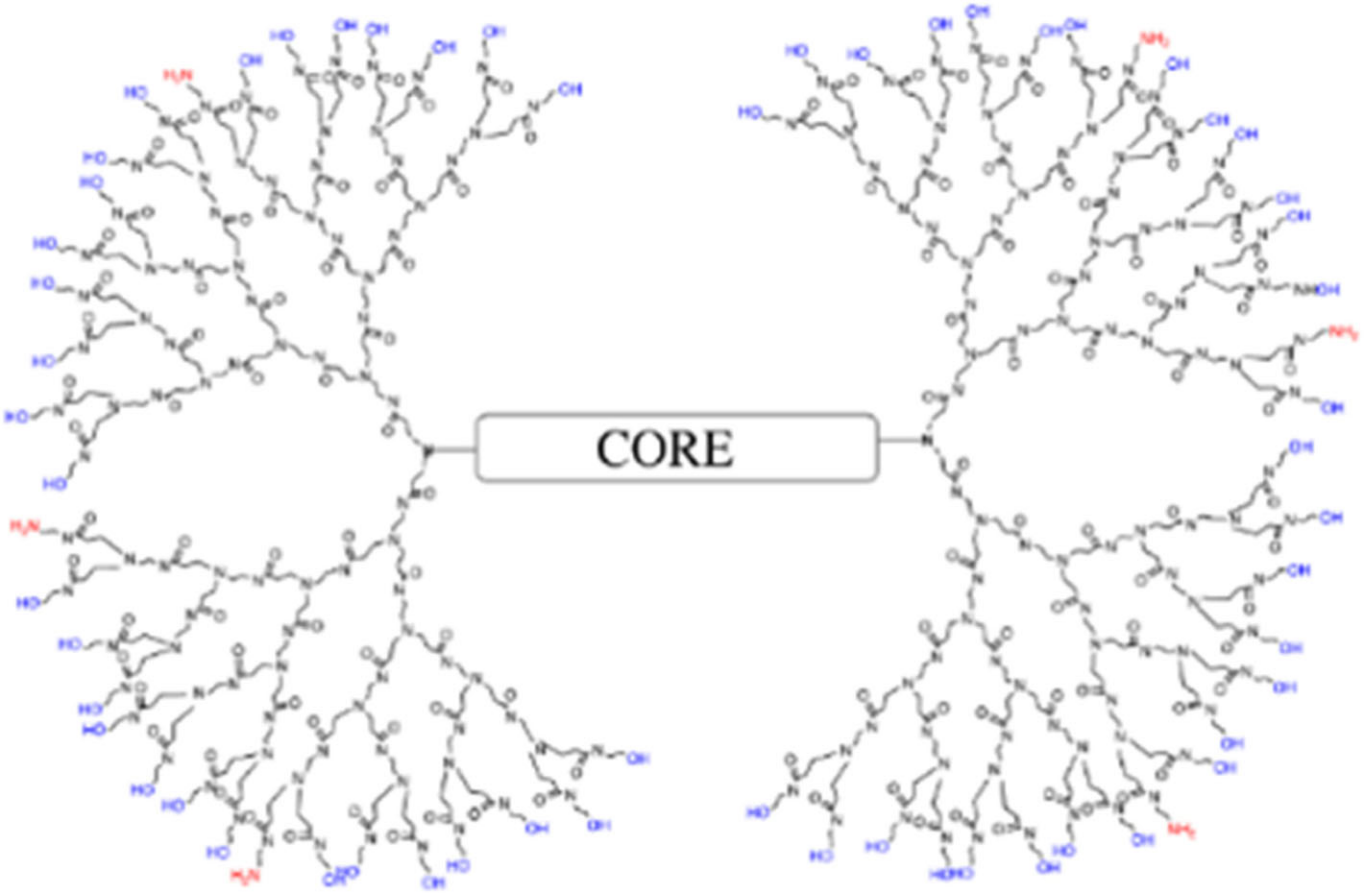
Schematic representation of G4 PAMAM dendrimer. The surface of the dendrimer is composed of 90% –OH and 10% –NH_2_ surface

The D were synthesized and labeled with FITC and Cy5.5 as previously described, and the purity of the dendrimers was verified by acidic polyacrylamide gel electrophoresis (PAGE) and RP-HPLC [19]. Acidic polyacrylamide gel electrophoresis showed one major band stained with Coomassie Blue while RP-HPLC showed one major band. The high purity of the mixed-surface dendrimer was possible since excess reagent was used in its synthesis, similar to the synthesis of traditional PAMAM dendrimers (Fig. 1).

### In vitro

**Fig. 2:**
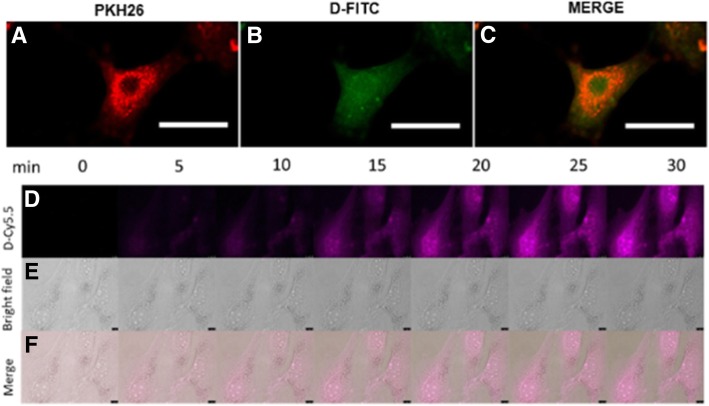
Labeling and uptake of D-FITC and D-Cy5.5 by BM-MSCs. (**a**) PKH26-labeled MSCs and (**b**) D-FITC-labeled MSCs are co-localized (**c**) with PKH26 localized in the cytoplasm, and D-FITC distributed relatively diffusely throughout the MSC. (**d**) Real-time uptake of the D-Cy5.5 by the BM-MSCs at regular intervals of time up to 30 min. (**e**) Bright field showing the BM-MSCs (**f**) co-localizing with the D-Cy5.5-labeled MSCs (scale bar 20 μm)

Our results showed that the P7 BM-MSCs successfully took up the D-FITC at 4 mg/mL when incubated for 30 min, which was found to be an optimal concentration and incubation time for cellular uptake. After entry into the cells, the co-localization between the cytoplasmic stain and the D-FITC are shown in Fig. 2a, b, and c.

### Real-time uptake of D-Cy5.5 into MSCs

A 30-min real-time imaging experiment showed that the D-Cy5.5 (final concentration 4 mg/mL) enters the cells at around 5 min and the intensity of the fluorescence from the dendrimers increases with time (Fig. 2d). A 24-h real-time imaging experiment showed that the fluorescence intensity of the dendrimer-Cy5.5 peaked at 1 h, after which it decreased to a minimal level by 15 h (figure not shown).

These outcomes show that the type of fluorescent dye that is attached to the dendrimers does not affect their uptake by the cells.

### The cells proliferated and retained D-FITC up to 4 passages following uptake

**Fig. 3:**
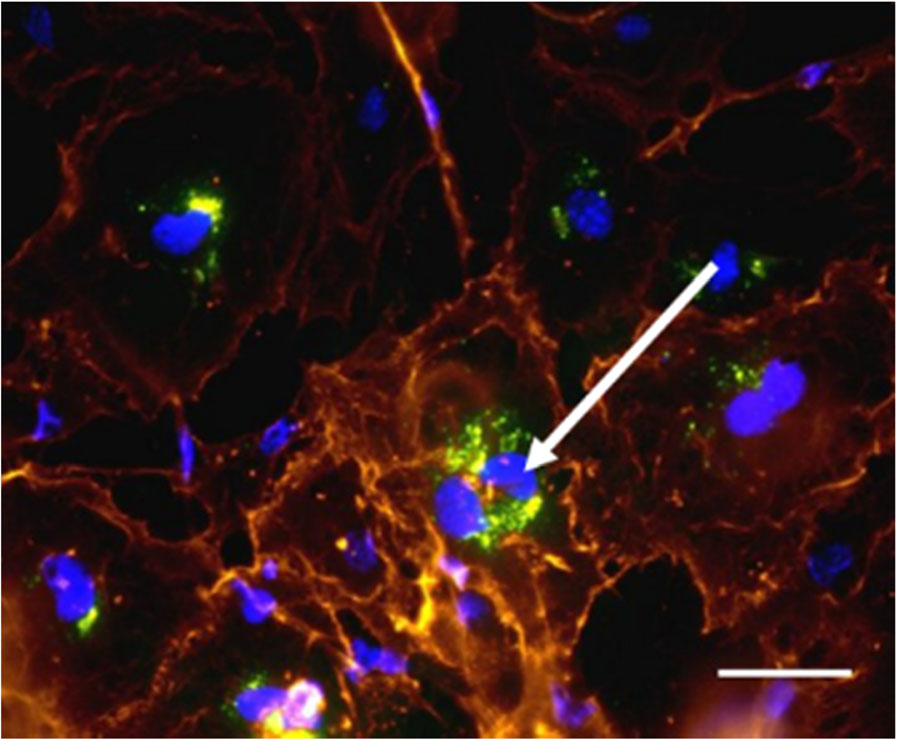
BM-MSCs retain their stemness following D-FITC uptake. BM-MSCs labeled with Hoechst (blue) and D-FITC (green, arrow) expressed Sca-1 in the cytoplasm, a cell marker specific to mouse BM-MSCs (brown), which shows that the stemness of the MSCs is retained after dendrimer uptake. Scale bar 20 μm

Once the MSCs have taken up the D-FITC (as shown in Fig. 3), the cells were passaged each time they attained 80% confluency. Our results showed that the D-FITC nanomolecules were retained within MSCs for up to four passages following their uptake (data not shown). This indicates that dendrimers are likely staying within the parent cell during replication. As MSCs replicate, fluorescence may diminish, as the D-FITC are localizing within the two daughter cells that emerge from each parent cell.

### MSCs retain their stemness following uptake of D-FITC

MSCs were analyzed between every passage for the stemness marker, Sca-1, following their uptake of D-FITC and expressed this specific marker in their cytoplasm. The MSCs were pre-labeled with Hoechst. Our results showed co-localization between Hoechst stain, D-FITC, and Sca-1 marker confirming that the MSCs that have taken up the D-FITC retain their stemness **(**Fig. 3**)**.

### Analysis of leakiness of the dendrimers following cell proliferation

UV-visible analysis of cell culture media collected between every passage following D-FITC uptake showed the absence of the 494 nm FITC peak, indicating that there was no leakage of the dendrimer from the labeled cells.

### Differentiation

**Fig. 4:**
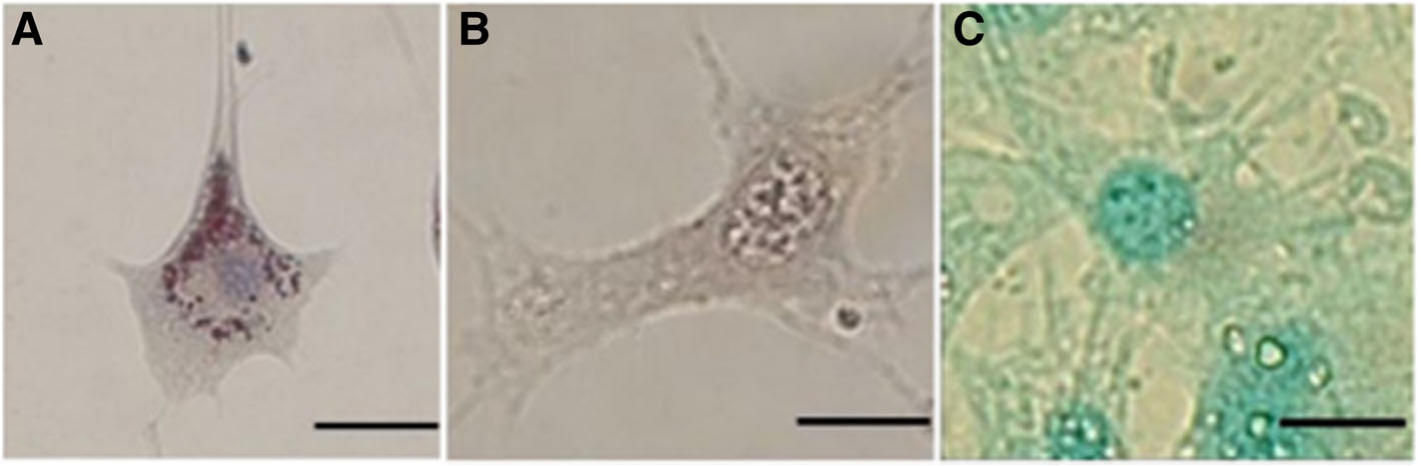
Tri-lineage differentiation of BM-MSCs following D-Cy5.5 uptake. (**a**) MSCs differentiated to become adipocytes were stained with Oil Red O and showed positive red lipid droplets and blue nucleus for MSCs transfected with Cy5.5 G4-90/10 dendrimer. (**b**) MSCs differentiated to become osteoblast were stained with Alizarin Red and showed positive orange red extracellular calcium deposits for MSCs transfected with D-Cy5.5 and (**c**) MSCs differentiated into chondrocytes were stained with Alcian Blue and showed positive blue-colored sulfonated mucosubstance following transfection with D-Cy5.5. Scale bar 20 μm

MSCs that were labeled with D-Cy5.5 showed differentiation for all three lineages in a similar fashion as the control MSCs that were not labeled with dendrimer. MSCs both with and without the dendrimer that differentiated into (1) adipocytes stained with Oil Red O to reveal red lipid droplets juxtaposed to its blue-stained nucleus (Fig. 4a), (2) osteoblasts stained with Alizarin red and demonstrated to show calcium deposits (Fig. 4b), and (3) chondrocytes stained with Alcian blue and demonstrated blue sulfonated mucosubstances (Fig. 4c). The positive staining for all three differentiated lineages indicates that MSCs maintain their innate ability to differentiate, despite being transfected with dendrimers. Undifferentiated cells with and without dendrimer (D-Cy5.5) showed absence of staining with Alizarin red, Oil Red O, or Alcian Blue (data not shown).

### Genetically modified MSCs and their characterization

**Fig. 5:**
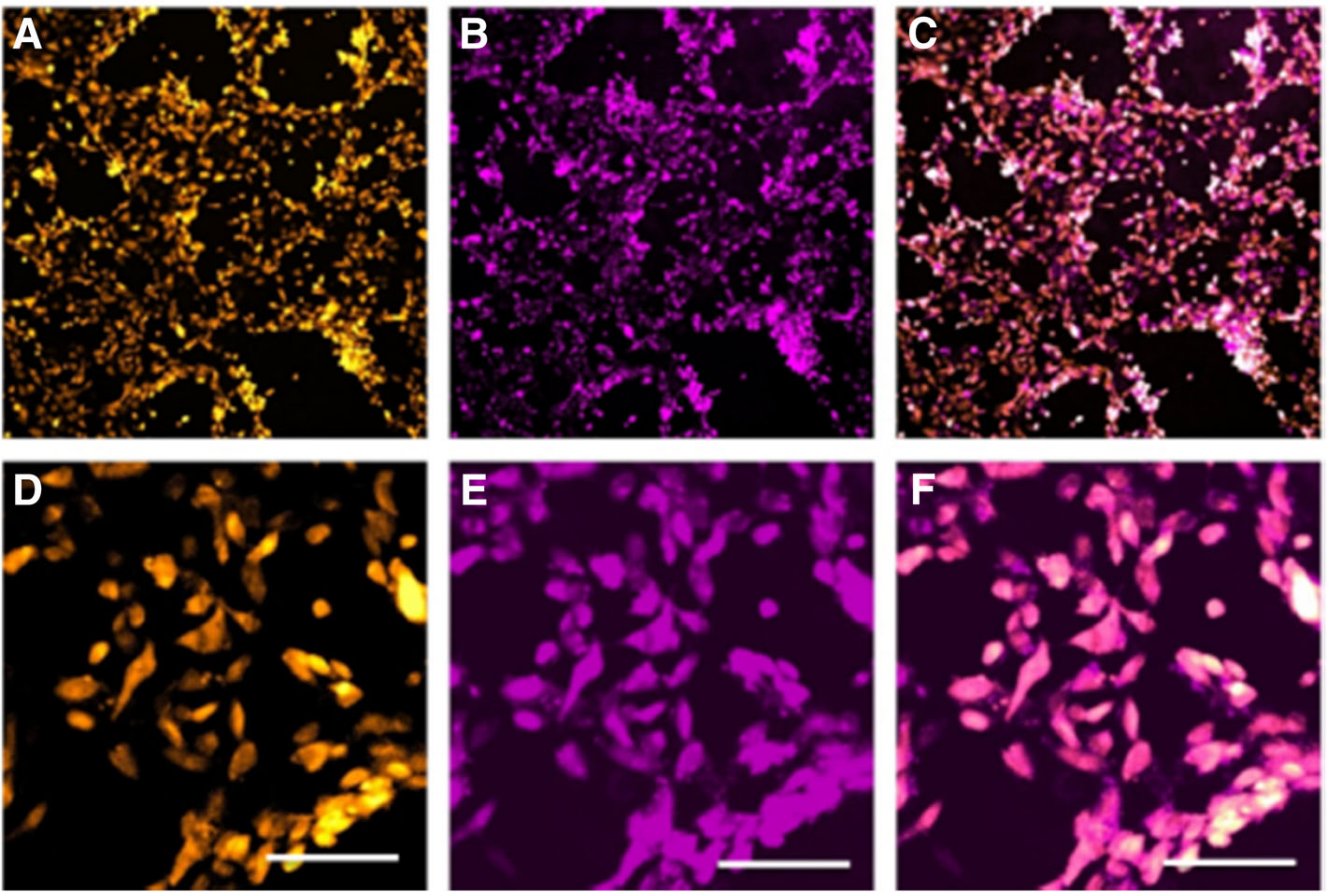
Labeling and uptake of D-FITC by tdT expressing BM-MSCs (**a**) BM-MSCs expressing tdT (**b**) that have taken up D-Cy5.5. (**c**) Colocalized within the mesenchymal stem cells. The zoomed in images below (**d**–**f**) correspond with the above images (**a**–**c**; scale bar 100 μm)

The MSCs were successfully modified to express tdT. The modified and control MSCs were both able to successfully differentiate into osteoblasts, chondrocytes, and adipocytes, as evidenced by their respective staining. This indicates that genetically modifying the MSCs does not change their properties (data not shown). Furthermore, these cells successfully took up the D-Cy5.5 at the specified concentrations mentioned above (Fig. 5).

### In vivo

**Fig. 6:**
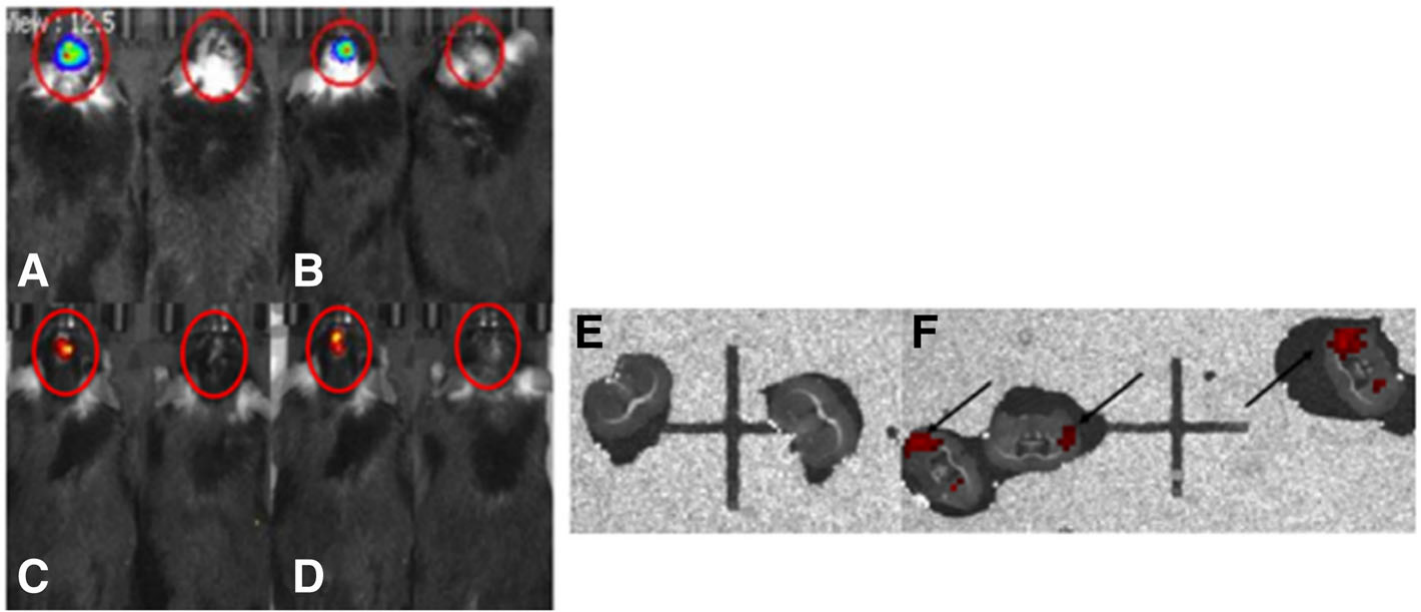
Tracking of the transplanted labeled stem cells using IVIS in vivo and ex vivo: D-Cy5.5-labeled MSCs expressing tdT can be observed for up to 2 weeks post-transplantation by the In vivo Imaging System. **a**, **b** Luciferase bioluminescence during 1 week (**a**) and 2 weeks (**b**) following transplantation. **c**, **d** Cy5.5 fluorescence during 1 (**c**) and 2 (**d**) weeks following transplantation. Control animals (right of each image) that received HBSS did not produce any signal. (**e**) Sections of the brain that received HBSS do not show any fluorescence. (**f**) Sections of the brain that received D-Cy5.5-labeled MSCs show fluorescence signal from D-Cy5.5 at the striatum (arrows)

The bioluminescence from the luciferase in the MSCs and the fluorescence from D-Cy5.5 was tracked in the mice following transplantations of MSCs labeled with D-Cy5.5 by in vivo imaging at 1- and 2-week time points. The counts of bioluminescent photons emitted from the dendrimer-labeled MSCs expressing tdTomato at week 1 was found to be at 1.505e+05, while at week 2, the photon count was found to be at 4.95e+04 (Fig. 6a, b). The fluorescent photon count of the same labeled MSCs at week 1 was found to be 9.714e+03, while at week 2, the photon count was found to be 7.662e+03 (Fig. 6c, d).

### Ex vivo imaging

Ex vivo imaging of the brain sections on the IVIS showed fluorescence from the D-Cy5.5 in the striatum compared to the striatal sections that received HBSS (Fig. 6e, f).

### Microscope imaging

**Fig. 7:**
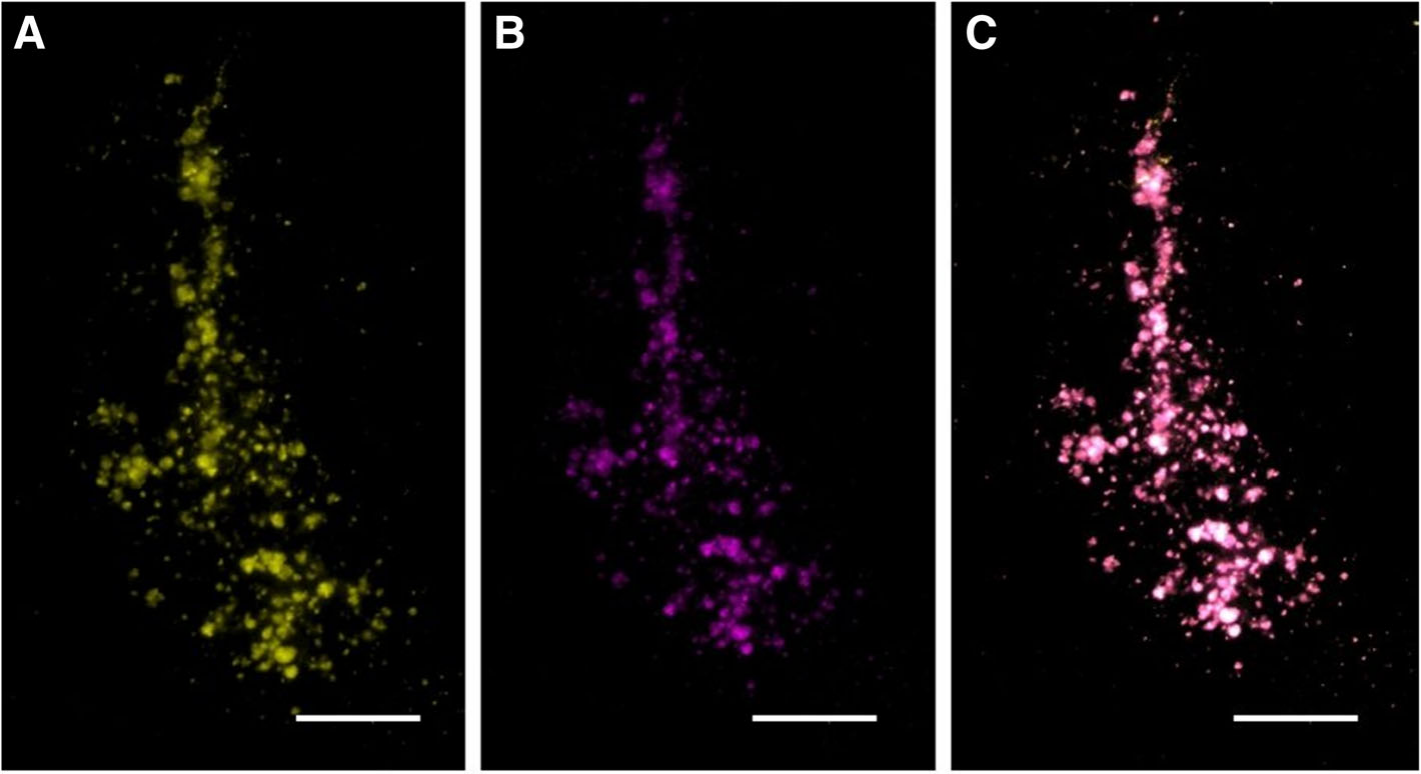
Presence of MSCs labeled with D-Cy5.5 in the striatum. (**a**) tdT MSCs in the striatum and (**b**) D-Cy5.5-labeled MSCs are colocalized, which is shown in merged image (**c**). Images were taken using the Zeiss Axio Imager M1 microscope (scale bar 100 μm)

Two weeks following transplantations, the brain slices were imaged under a fluorescence microscope (Zeiss Axio Imager M1 microscope). Our results showed that there was co-localization between tdT and Cy5.5, proving that the dendrimers remained in the tdT MSCs following transplantations (Fig. 7).

## Discussion

We have shown that safe, biocompatible PAMAM mixed-surface dendrimers can be used for labeling MSCs in vitro and in vivo without changing the innate properties of these cells. We previously showed that the G4-90/10 dendrimers are much safer than G4 amine-terminated dendrimers due to a tenfold reduction in their surface amines. There were sufficient surface amines (~ 5–6 per dendrimer) to readily tag the G4 mixed-surface dendrimer with fluorescent dyes (FITC and Cy5.5) using conventional fluorescent reagents made for proteins (FITC and NHS-Cy5.5). We also proved that these dendrimers do not leak out of the cells during cell proliferation following dendrimer uptake. Therefore, once the labeled cells are transplanted into the brain, the dendrimers are retained as long as the cells survive. However, an important aspect to be noted is the senescence associated with these stem cells, which will later lead to their death. Alessio and colleagues have shown that low dose of radiation can lead to senescence [22]. Senescence of dendrimer-labeled MSCs needs further investigation. Since the dendrimers do not leak out of the cells, they are retained within the MSCs and do not label surrounding host cells, thereby avoiding false positives that would occur if the label was transferred to host cells. Given that MSCs can differentiate into different lineages including osteoblasts, adipocytes, and chondrocytes, we assessed whether this differentiation might be affected by the dendrimer label. Previous studies have shown that introduction of certain nanoparticles to MSCs affects their differentiation. For example, nanophase hydroxyapatite, HA-PLGA nanocomposites, and iron oxide nanoparticles (IONP) have been shown to induce osteogenic differentiation from human MSCs [23]. PLLA-hydroxyapatite nanocomposites also induce chondrogenic differentiation in MSCs, while graphene quantum dots (GQD) have been shown to enhance differentiation of MSCs into osteoblasts and adipocytes [24, 25]. Though this can be useful when treatments require for the differentiation into a specific cell line, these nanoparticles cannot be used for labeling if it is important for the MSCs to retain their “stemness.” Our results clearly show that our mixed-surface dendrimers label MSCs without altering their inherent properties of differentiation. Our experiments showed that MSCs were able to differentiate, with and without dendrimers in them. This is critical if this label is used in MSCs that requires retention of their regenerative properties. Our findings also clearly show that dendrimer-labeled MSCs can be used for differentiation into any cell line that is needed for the purpose of regenerative medicine.

We also looked at the ability of fluorescent-labeled dendrimers to track MSCs in the brain. Both the Luc2-dTom and Cy5.5-labeled dendrimers were able to co-localize in vitro. It was found that the dendrimer-labeled MSCs were proficient in tracking the MSCs post-transplantation in the brain. At 1-week and 2-week post-transplantation, images of the brain taken via the IVIS revealed bioluminescence of the luc2-dTom the fluorescence of the D-Cy5.5. Upon brain extraction, co-labeling of tdT and D-Cy5.5 was also observed. Mixed-surface G4 PAMAM dendrimers thus offer a new tool to safely track transplanted cells in vivo*.*

## Conclusions

The G4 mixed-surface dendrimer was designed and synthesized to have a biocompatible surface composed predominantly (90%) of hydroxyl groups. This is necessary since G4 dendrimers with pure amine surface are known to be toxic to cells. The few amines available on the mixed-surface dendrimer (10% or 6 per dendrimer) were necessary for the attachment of fluorescent dyes for imaging purposes. This study shows that these nanomolecules were readily taken up by mesenchymal stem cells to form dendrimer-labeled MSCs. The stemness, proliferation, and differentiation properties of dendrimer-labeled MSCs remain unchanged compared to unlabeled MSCs. The labeled MSCs retained the fluorescent-tagged dendrimers even up to four passages in vitro. Intracranial administration of the dendrimer-labeled MSCs in mice showed that the cells can be imaged and tracked for 2 weeks using an in vivo imaging system. Overall, this study demonstrates the successful utilization of mixed-surface PAMAM dendrimers as a safe and effective tool for labeling stem cells, and possibly, many other cell types, both for in vivo and ex vivo purposes.

### Future directions

Our future plans are to utilize these dendrimer-labeled MSCs in studies designed to test their efficacy in treating neurodegenerative processes in animal models of Huntington’s, Parkinson’s, and Alzheimer’s diseases.

## Abbreviations

BM: Bone marrow
BM-MSC: Bone marrow-derived mesenchymal stem cell
MSC: Mesenchymal stem cell
BDNF: Brain-derived neurotrophic factor
bFGF: Basic fibroblast growth factor
Cy5.5: Cyanine 5.5
DMEM: Dulbecco’s modified Eagle medium
FBS: Fetal bovine serum
FITC: Fluorescein isothiocyanate
GQD: Graphene quantum dots
HBSS: Hank’s balanced salt solution
HS: Horse serum
IVIS: In vivo imaging system
Luc2: Luciferase
NHS: *N*-Hydroxysuccinimide
PAGE: Polyacrylamide gel electrophoresis
PAMAM: Polyaminoamine
PBS: Phosphate-buffered saline
PB: Phosphate buffer
PFA: Paraformaldehyde
PLGA: Poly(d,l-lactide-co-glycolide)
PLL: Poly-l-lysine
RP-HPLC: Reversed-phase high-performance liquid chromatography
Sca-1: Stem cell antigen-1
tDT: tdTomato
UV: Ultraviolet
